# Seasonal Variations of Complete Blood Count and Inflammatory Biomarkers in the US Population - Analysis of NHANES Data

**DOI:** 10.1371/journal.pone.0142382

**Published:** 2015-11-06

**Authors:** Bian Liu, Emanuela Taioli

**Affiliations:** Department of Population Health Science and Policy, Icahn School of Medicine at Mount Sinai, and Institute for Translational Epidemiology, New York, New York, United States of America; University of Nebraska Medical center, UNITED STATES

## Abstract

**Background:**

Recent studies reported seasonal differences in gene expression in white blood cells, adipose tissue, and inflammatory biomarkers of the immune system. There is no data on the seasonal variations of these biomarkers in the US general population of both children and adults. Then aim of this study is to explore the seasonal trends in complete blood count (CBC), and C-reactive protein (CRP) in a large non-institutionalized US population.

**Methods:**

Seven cross-sectional data collected in the National Health and Nutrition Examination Survey (NHANES) during 1999–2012 were aggregated; participants reporting recent use of prescribed steroids, chemotherapy, immunomodulators and antibiotics were excluded. Linear regression models were used to compare levels of CBC and CRP between winter-spring (November-April) and summer-fall (May-October), adjusting for demographics, personal behavioral factors, and chronic disease conditions.

**Results:**

A total of 27,478 children and 36,644 adults (≥18 years) were included in the study. Levels of neutrophils, white blood cell count (WBC), and CRP were higher in winter-spring than summer-fall (p≤0.05). Red blood cell components were lower in winter-spring than in summer-fall, while the opposite was seen for platelets.

**Conclusions:**

This large population-based study found notable seasonal variations in blood cell composition and inflammatory biomarkers, with a more pro-inflammatory immune system seen in winter-spring than summer-fall. The red blood cell patterns could have implications for the observed cardio-vascular seasonality.

## Introduction

Seasonal patterns in human health, such as seasonal affective disorder [1], arthritis [2], blood pressure [3], as well as cardiovascular and respiratory morbidity and mortality [4–7] have been recognized for a long time. While the molecular and cellular mechanism for these seasonal variations are not clear, recent studies suggest that gene expression periodicity in white blood cells (WBC), adipose tissue, and inflammatory biomarkers of the immune system [8–10] could be one of the underlying explanations. Both longitudinal and cross-sectional studies have suggested seasonal variations in blood cellular component and inflammatory biomarkers such as C-reactive proteins (CRP) in family studies [10], patients populations [8,10], or in occupational setting [11]. To our knowledge, there is no data on the seasonal variations of these biomarkers on both children and adults in a representative large US general population. The aim of this study is to explore the seasonal trends in complete blood count (CBC) and CRP in the US population. Given the previous results that point to a pro-inflammatory immune system and elevated mortality and morbidity during cold seasons [2–10,12–14], we hypothesized increased levels of inflammatory biomarkers in winter-spring in comparison to summer-fall seasons.

## Materials and Methods

### Study subjects

The study participants were from the National Health and Nutrition Examination Survey (NHANES) conducted by the US National Center for Health Statistics (NCHS) of the Centers for Disease Control and Prevention (CDC). The survey assesses a variety of health issues and nutritional status of the US non-institutionalized civilian population based on a combination of questionnaires and physical examinations administered in homes and mobile examination centers. The participants were selected using a stratified, multi-stage, probability-cluster design in order to provide a national representative sample. The ethical, privacy, and confidentiality protocols of NHANES were developed and reviewed in compliance with the policies for protection of human research subjects developed by the US Department of Health and Human Services [15]. Sample persons were informed of the NHANES survey process and their rights as a participant by interviewers and by written materials. A parent or guardian gave permission for minors to participate, and children aged 7–17 also provided documented assent prior to participating, while emancipated minor did not need parental permission. The consent forms were presented in a specific order to ensure all necessary signatures were captured, and documented signed consent was obtained. The NCHS Research Ethics Review Board (ERC) reviewed and approved NHANES protocols annually [15].

Seven cross-sectional surveys (1999–2012) were extracted from the NHANES publically available database and were aggregated. Of the 71916 participants, 4777 (6.7%) were excluded due to reported recent use of steroids, chemotherapy, immunomodulators and antibiotics, and 3017 (4.2%) due to lack of information on the time period the examination was performed. The final study population consisted of 64122 participants, among which 54855 had complete blood count (CBC) data, and 44434 had C-reactive protein (CRP) data, which was only available for the period of 1999–2010.

### Biosamples and covariates

The time when blood samples were drawn was recorded in the public-use files as either winter-spring (November-April) or summer-fall (May-October) seasons. Slightly more samples were taken during the summer-fall than winter-spring (53% vs 47%). The main response variables of interest were CRP and 13 types of cellular blood composition: hemoglobin (HGB), mean cell hemoglobin (MCH), mean cell volume (MCV), hematocrit (HCT), red cell count (RBC), platelet count (PLT), basophils number (BAS), eosinophils number (EOS), monocyte number (Mon), segmented neutrophils number (Neu), lymphocyte number (Lym), neutrophil/lymphocyte ratio (NLR), and white blood cell count (WBC). CRP was quantified by latex-enhanced nephelometry, and CBC analysis was performed on the Coulter^®^ method, following the NHANES quality control and quality assurance protocols [15].

Covariates extracted included demographics: age, sex, education (<, =, or > high school level), race (whites or others), family income to poverty ratio (PIR, ≤ or >1); personal behavior factors: smoking history (yes/no) and alcohol consumption (yes/no); body mass index (BMI); and self-reported history of 16 major chronic diseases (yes/no): asthma, arthritis, congestive heart failure, coronary heart disease, angina pectoris, heart attack, stroke, emphysema, overweight, chronic bronchitis, liver diseases, thyroid problem, cancer, diabetes, high cholesterols, and hypertension.

### Statistical Analysis

Seasonal differences in CBC and CRP between winter-spring and summer-fall were first compared using Wilcoxon tests separately for children and adults (≥18 years). Seasonal trends were further investigated using linear regression models, applying sampling weights to account for the complex sampling NHANES design [16], and using natural log transformed CBC, CRP, and BMI. The (log(x+1) transformation was used for BAS, EOS, and Mon to include the zero counts. Models were run separately on children and adults, adjusting for covariates (age, sex, race, PIR, BMI, and chronic disease status (yes to any or none of the 16 diseases). For adults, additional covariates (education, smoking, and alcohol consumption status) were included in the model. Sensitivity analyses were conducted on the healthy sub-group consisting of those had none of the chronic diseases. Statistical analyses were performed using SAS (version 9.4, Cary, NC).

## Results

### Study population characteristics

Overall, 27,478 children and 36,644 adults (≥18 years) were included in the study. The weighted mean age (± standard error, SE) was 8.7±0.04 and 45.5±0.2 years for children and adults, respectively (Table 1). Male participants represented 51% and 49% of the children and adult groups, respectively. The majority (>75%) of the study participants had family income to poverty ratio (PIR) greater than one. Approximately 47% of the adults had smoked at least 100 cigarettes in their entire lives, and 74% of participants had at least 12 alcohol drinks yearly. More than half (62%) of the adults and 14.5% of the children had at least one of the sixteen self-reported chronic conditions.

**Table 1:** Weighted characteristics of the study population- NHANES (1999–2012).

		**< 18 years**			**18+ years**		
		**Number**	**%**	**SE**	**Number**	**%**	**SE**
**Sex**	**Male**	13946	51.1	0.4	17887	48.7	0.3
	**Female**	13532	48.9	0.4	18757	51.3	0.3
**Race**	**White**	7498	57.0	1.4	16460	68.9	1.2
	**Others**	19980	43.0	1.4	20184	31.1	1.2
**Poverty Income Ratio**	**PIR≤1**	9043	24.5	0.7	7482	15.1	0.5
	**PIR>1**	16384	75.5	0.7	25962	84.9	0.5
**Education**	**<High School**				10136	19.6	0.5
	**High School**				7877	24.5	0.5
	**>High School**				15642	55.9	0.8
**Smoking History**	**Ever Smoker**				15697	47.3	0.6
	**Never Smoker**				17984	52.7	0.6
**Alcohol Consumption**	**Current drinker**				21462	74.4	0.7
	**Non-current drinker**				9315	25.6	0.7
**Chronic Diseases**	**Yes**	3778	14.5	0.4	22707	62.4	0.6
	**No**	23700	85.5	0.4	13937	37.6	0.6
		**N**	**Mean**	**Range**	**N**	**Mean**	**Range**
**Age**	(years)	27478	8.7	0–17	36644	45.5	18–85
**BMI**	(kg/m^2^)	22318	19.1	8–62.7	35798	27.6	13.2–130.2
**Hemoglobin**	(g/dL)	20317	13.4*	6.4–19	34671	14.3*	5.8–19.7
**Mean cell hemoglobin**	(pg)	20317	28.9	14.9–42.8	34671	30.6*	14.7–60.8
**Mean cell volume**	(fL)	20317	84.4	50.7–106.5	34671	89.7*	50.5–120.6
**Hematocrit**	(%)	20317	39.2	20.8–55.5	34671	41.9	19.7–59.9
**Red cell count**	(10^6^ cells/uL)	20317	4.6*	2.5–7.3	34671	4.7	2.3–9.2
**Platelet count**	(10^3^ cells/uL)	20316	294.7*	13–1000	34670	251.9*	4–1000
**Basophils number**	(10^3^ cells/uL)	20305	0.04	0–2	34511	0.04	0–4.7
**Eosinophils number**	(10^3^ cells/uL)	20305	0.2	0–5.3	34511	0.2	0–8.4
**Monocyte number**	(10^3^ cells/uL)	20305	0.6*	0.1–3.8	34550	0.5*	0–10.2
**Segmented neutrophils number**	(10^3^ cells/uL)	20305	3.3*	0.2–18.5	34551	4.0	0.1–83.1
**Lymphocyte number**	(10^3^ cells/uL)	20305	2.6	0.4–15.5	34551	2.0*	0.4–89.7
**Neutrophil to Lymphocyte ratio**		20305	1.3*	0.04–17.1	34551	2.0*	0.009–28.7
**White blood cell count**	(10^3^ cells/uL)	20316	7.0*	1.4–26.3	34670	6.9*	1.5–100
**C-reactive protein**	(mg/dL)	14994	0.04*	0.01–13.6	29440	0.2*	0.01–29.6

**Note:** Geometric means were calculated (except for Age). SE = standard error.

*, Seasonal differences (winter-spring vs summer-fall, Wilcoxon tests) were statistically significant at *p*≤0.001 level. Statistics are based on weighted estimates accounting for the complex sampling design of NHANES. Percentages are not calculated directly on the absolute numbers presented in the table.

The overall geometric means of WBC and neutrophils number were similar between children and adults, while CRP was lower in children (0.04 mg/dL) than in adults (0.2 mg/dL) (Table 1).

### Seasonal variation

**Fig 1:**
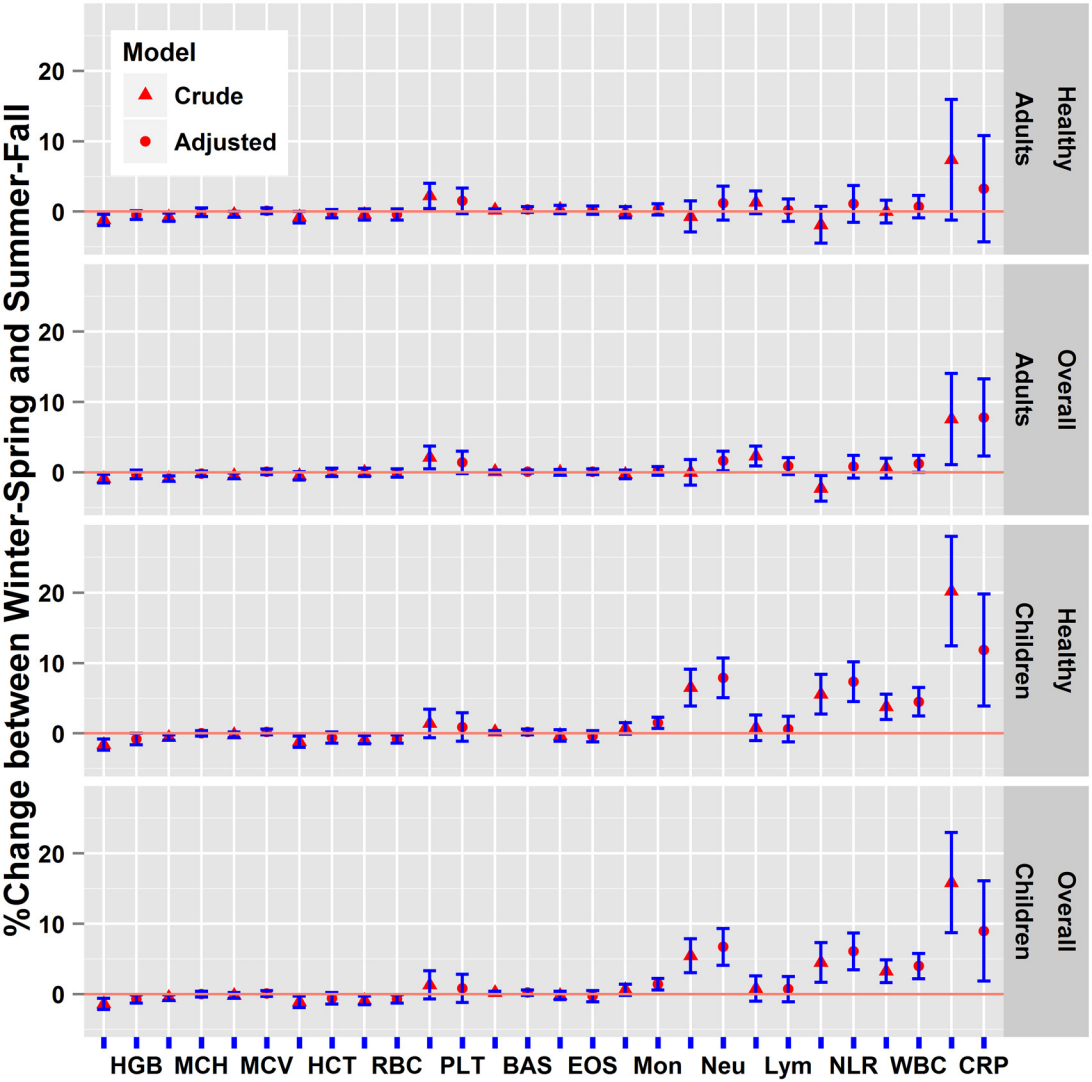
Differences in complete blood count and C-reactive protein levels between winter-spring and summer-fall (reference) seasons based on regression coefficients (±2*standard error) from crude and adjusted models, NHANES (1999–2012). **Note:** %change above zero indicates higher winter-spring than summer-fall levels, while below zero indicates lower winter-spring than summer-fall levels. Regressions were run separately for children and adults (18+ years) populations. Analyses were conducted separately for the overall population and the healthy group, defined as those without any of the 16 self-reported chronic diseases. Regression models, log(biomarkers) = f(season, covariates), were adjusted for age, sex, race, poverty income ratio, and body mass index, and chronic disease status. Additional covariates: education, smoking, and alcohol consumption were adjusted for the adult population. BAS = Basophils number, EOS = Eosinophils number, HCT = Hematocrit, HGB = Hemoglobin, Lym = Lymphocyte number, MCH = Mean cell hemoglobin, MCV = Mean cell volume, Mon = Monocyte number, Neu = Segmented neutrophils number, PLT = Platelet count, RBC = Red cell count, WBC = White blood cell count, NLR = Neu/Lym ratio, CRP = C-reactive protein.

Among adults, all CBC variables showed statistically significant seasonal differences at *p*≤0.001 (Table 1), except for BAS (*p* = 0.9), EOS (*p* = 0.07), Neu (*p* = 0.07), RBC (*p* = 0.3), and HCT (*p* = 0.003); higher levels of Neu, WBC, and CRP were observed in winter-spring than in summer-fall. These three variables became non-significant among the subset (~40% of the total sample) of healthy subjects who did not report any chronic diseases. In the crude models for both overall and healthy adults, HGB and MCH were statistically lower in winter-spring than summer-fall, and PLT were higher in winter-spring than summer-fall (Fig 1). While these seasonal trends remained in the adjusted models, the effects did not reach the statistical significance at *p*<0.05.

Among children, all the CBC variables showed statistically significant seasonal variation at *p*≤0.001 (Table 1), except for BAS (*p* = 0.4), EOS (*p* = 0.006), Lym (*p* = 0.3), MCH (*p* = 0.9), MCV (*p* = 0.03), and HCT (*p* = 0.003). Higher levels of Mon, Neu, NLR, WBC, and CRP were found in winter-spring than in summer-fall (Fig 1), while HGB and RBC were lower in winter-spring than summer-fall. Similar results were seen in the subset (~85% of the total sample) of healthy children without any chronic diseases. In the crude models, HCT and MCH were significantly lower in winter-spring than in summer-fall, but the results became non-significant in the adjusted models.

The regression coefficients (Table 2) for the three biomarkers that showed statistically significant seasonal heterogeneities in both children and adults, indicate that the magnitude of seasonal differences in CRP was similar for both children (9%) and adults (8%), while for neutrophils and WBC, larger seasonal differences were seen among children (4–7%) than among adults (1–2%). Several covariates in the models were also significant predictors of the biomarker levels (Table 2). For example, BMI was positively associated with levels of WBC, Neu, and CRP in both children and adults. Females tend to have higher levels of the inflammatory indicators than males. The gender difference was pronounced among adults for CRP, which was 44% higher in females than males with all the other variables held constant. Ever smokers had higher inflammatory levels (~9–21%) than non-smokers. Comparing to whites, non-white tended to have higher CRP (11% and 4% in children and adults, respectively), while the reverse was true for Neu and WBC levels (10% and 5% lower in children and adults, respectively). Having a better socioeconomic status, such as having higher education levels or above poverty incomes, were associated with decreased levels in WBC, Neu, and CRP.

**Table 2:** Regression coefficients for CRP, Neutrophils (Neu), and White Blood Cell (WBC) between winter-spring and summer-fall in children and adults (18+ years), NHANES (1999–2012).

Comparison	<18 years			18+ years		
	CRP	Neu	WBC	CRP	Neu	WBC
**Winter-Spring vs Summer-Fall**	0.09*	0.07***	0.04***	0.07**	0.02*	0.01*
**Female vs male**	0.07**	0.07***	0.04***	0.36***	0.04***	0.03***
**Other race vs white**	0.11**	-0.10***	-0.05***	0.04*	-0.11***	-0.05***
**Education**				-0.08***	-0.03***	-0.03***
**PIR>1 vs PIR≤1**	-0.07	-0.04**	-0.02	-0.12***	-0.05***	-0.04***
**Smoker vs non smoker**				0.19***	0.09***	0.09***
**Drinker vs non drinker**				-0.05*	-0.002	-0.01
**Chronic disease vs no**	0.05	-0.01	0.01	0.11***	0.002	0.004
**Age**	-0.07***	-0.001	-0.02***	0.01***	-0.002***	-0.002***
**BMI**	3.12***	0.46***	0.32***	2.63***	0.29***	0.25***

**Note:** Results from regression models on the overall population, adjusting for age, sex, race (white or non-white), poverty income ratio (PIR>1 or ≤1), and BMI, and chronic disease status (self-reported versus no reporting of 16 major chronic diseases: asthma, arthritis, congestive heart failure, coronary heart disease, angina pectoris, heart attack, stroke, emphysema, overweight, chronic bronchitis, liver diseases, thyroid problem, cancer, diabetes, high cholesterols, and hypertension). Additional covariates: education (<, =, and > high school), smoking, and alcohol consumption were adjusted in the adult data set. Levels of Neu, WBC, CRP, and BMI were natural log transformed. Neu = Segmented neutrophils number, WBC = White blood cell count, CRP = C-reactive protein, Ref = Reference level. Significance levels:

*, *p*<0.05;

**, *p*≤0.01;

***, *p*≤0.001.

## Discussion

This study confirms the existence of seasonal variations in blood inflammatory biomarkers, such as CRP, WBC, and neutrophils in both adult and children at the population-level, with a more pro-inflammatory immune system activation seen in winter-spring than summer-fall. High pro-inflammatory blood markers in the winter-spring could simply reflect an innate defense mechanism against infectious diseases such as cold and flu, whose epidemics generally occur in the winter season. However, the same markers could be partly responsible for the observed seasonality of diseases that have a marked inflammatory milieu, such as atherosclerosis, as well as auto-immune conditions such as rheumatoid arthritis and type I diabetes [17]. Apart from seasonal changes in human physiology in response to diseases and environment (e.g. UV radiation and temperature), the observed seasonal variation could also reflect the effects of other factors. For example, studies have shown seasonal variations in physical activity levels, which have been shown to influence CRP levels [18], tend to peak in the summer and reach a low in the winter [19,20]. Seasonality of blood biomarkers, especially among adults, has been reported before with inconsistent results. Increased CRP in winter-spring observed in the NHANES data set were in agreement with the CRP seasonality reported by some [9,12,14], while borderline seasonal heterogeneity was also observed by others [13]. Discrepancies also exist in the seasonality of other blood count types. In this study, red blood cell related cellular components (i.e. RBC, HGB, MCH, MCV, and HCT) tended to have lower levels in the winter-spring than summer-fall in the univariate comparisons, in line with results reported by some [8], while in contrast with other observations [9–11,21]. Low RBC parameters could indirectly indicate low oxygen transport, as well as low ferritin, two components that could be involved in the know winter exacerbation of cardiovascular disease [22–25].

Platelet levels were statistically significantly (*p* = 0.01, crude models) higher in winter-spring than summer-fall in our study, similar to what was previously reported [8,26,27]. Platelets are important components of the thrombotic process, and the seasonal variability of stroke and other vascular events could reflect the season variability of platelets, together with a more pro-inflammatory blood environment [28–31].

While both BAS and EOS exhibited seasonality in Dopico *et al*. [9] study in a United Kingdom cohort of healthy volunteers, these two parameters did not show seasonal differences in the present study. Reasons for the discordant results include differences in the population characteristics, study design, and analytical methods. For example, our analysis was limited to a two-level seasonal variable while the analysis by Dopico *et al*. used a model based on monthly-recorded variables. Our models included a few more relevant covariates than the CRP models in Dopico *et al*., which adjusted for age and sex, as well as their interaction term. The two studies however were consistent in showing the seasonality of CRP. Dopico *et al*. [9] found seasonal variation of CRP levels in a population of hypertensive adults measured during the European winter. Our analyses on both generally healthy children and adults showed elevated CRP levels in winter-spring.

In our analysis we were able to present both crude and adjusted parameters for all the blood biomarkers studied. In the univariate comparisons, the majority of cellular components showed statistically significant seasonal differences. However, only a few remained significant in the multivariable analysis, where other factors known to be pro-inflammatory, such as age, sex, and BMI were more relevant than the seasonal effects in determining the variation of blood components. Variations of inflammation biomarkers among groups with different demographic and socioeconomic factors have been reported [32–34], although the underlying mechanisms are unclear. The adjustment of covariates takes into account, to a certain degree, the observed heterogeneity, while highlighting the independent contribution of seasonal effects in explaining the variation of inflammation biomarkers.

New from previous studies, which mainly focused on adult populations, the present analyses found a more pronounced seasonality among children than adults; consistent seasonality in Neu, NLR, WBC, and CRP were seen in children overall and in the healthy subset. It is unclear why children have a stronger seasonality in these serum biomarkers than adults. One possible explanation is that the developing immune system is more sensitive to seasonal fluctuations of environmental stressors (e.g. temperature and sunlight), compared to the mature immune systems. As higher levels of Neu, NLR, WBC, and CRP are suggestive of the presence of infection, the observed seasonality could imply higher rates of minor respiratory infections in winter-spring than summer-fall.

Our study has several strengths and limitations. The major strength is the use of a large, nationally representative non-institutionalized sample of US residents from 1999–2012. This comprehensive data set allows examination of seasonal variation in blood cellularity and CRP in both adult and children, and in relevant subgroups. We were also able to adjust for some of the already known important contributors to inflammation such as BMI, sex, age, medication, and chronic health conditions, while most of the previous investigations were based on crude univariate analyses. The study has limitations: the data were cross-sectional in nature, which only provided population-level estimates and prevented us from investigating intra-individual seasonal variations. Despite the fact that this is the first study looking at seasonal variation of blood cellularity adjusting for important covariates, there may still be other confounding factors which were not considered in our analysis (e.g. physical activity). In addition, the time resolution in this study is limited to two seasons, which could attenuate or miss more detailed seasonal fluctuations in biomarkers as seen in other studies where monthly data are available.

The present study has been successful in identifying season as a contributor to inflammatory biomarkers in the general population. While the overall seasonal effect is small compared to some of the major contributors of blood cellular variation such as BMI, it is significant and has implications in developing diagnostic biomarkers using peripheral whole blood. Our results also suggest that seasonality could be a potential confounder in biomarkers research utilizing whole blood samples. Further longitudinal research encompassing wide geographic locations is needed to better understand the effects of seasonal variations of inflammatory biomarkers in clinical evaluations of the severity and onset of human disease, particularly among children.
